# Identification and validation of a pyroptosis-related prognostic signature for thyroid cancer

**DOI:** 10.1186/s12935-021-02231-0

**Published:** 2021-10-09

**Authors:** Pu Wu, Jinyuan Shi, Wei Sun, Hao Zhang

**Affiliations:** Department of Thyroid Surgery, The First Hospital of China Medical University, Shenyang, China

**Keywords:** THCA, Pyroptosis, Prognostic signature, TMB, ssGSEA

## Abstract

**Background:**

Pyroptosis is a form of programmed cell death triggered by inflammasomes. However, the roles of pyroptosis-related genes in thyroid cancer (THCA) remain still unclear.

**Objective:**

This study aimed to construct a pyroptosis-related signature that could effectively predict THCA prognosis and survival.

**Methods:**

A LASSO Cox regression analysis was performed to build a prognostic model based on the expression profile of each pyroptosis-related gene. The predictive value of the prognostic model was validated in the internal cohort.

**Results:**

A pyroptosis-related signature consisting of four genes was constructed to predict THCA prognosis and all patients were classified into high- and low-risk groups. Patients with a high-risk score had a poorer overall survival (OS) than those in the low-risk group. The area under the curve (AUC) of the receiver operator characteristic (ROC) curves assessed and verified the predictive performance of this signature. Multivariate analysis showed the risk score was an independent prognostic factor. Tumor immune cell infiltration and immune status were significantly higher in low-risk groups, which indicated a better response to immune checkpoint inhibitors (ICIs). Of the four pyroptosis-related genes in the prognostic signature, qRT-PCR detected three of them with significantly differential expression in THCA tissues.

**Conclusion:**

In summary, our pyroptosis-related risk signature may have an effective predictive and prognostic capability in THCA. Our results provide a potential foundation for future studies of the relationship between pyroptosis and the immunotherapy response.

**Supplementary Information:**

The online version contains supplementary material available at 10.1186/s12935-021-02231-0.

## Introduction

Thyroid cancer (THCA) is the most common form of endocrine cancer worldwide and the number of THCA cases is icreasing [1]. Thyroid nodules are one of the most common clinical findings [2]. THCA can be divided into at least four subtypes, papillary thyroid carcinoma (PTC), follicular thyroid cancer (FTC), medullary thyroid cancer (MTC) and anaplastic thyroid cancer (ATC), based on the histological features of a THCA tumor. PTC is the most frequent histological subtype and accounts for more than 90% of all thyroid cancer cases [3]. The majority of PTC cases have a relatively better prognosis after surgery and ^131^I treatment compared to that of the other THCA subtypes [4]. However, cervical lymph node metastasis (LNM) is a potential factor that can lead to some patients suffering local recurrence and poor prognosis [5,6]. Therefore, it is important to construct novel prognostic models or find novel biomarkers which will make targeted therapies more feasible and improve the survival of patients with PTC.

Pyroptosis is a form of programmed cell death triggered by inflammasomes [7,8]. Pyroptosis has been found to be closely associated with some diseases like diabetic nephropathy and atherosclerosis. Some studies have found that pyroptosis is involved in the proliferation, invasion and metastasis of tumors. Pyroptosis results in cell swelling, plasma membrane lysis, chromatin fragmentation and the release of intracellular proinflammatory compounds. Pyroptosis is distinguished from other forms of programmed cell death morphologically although it shares certain similar characteristics with apoptosis. Generally, cells undergoing pyroptosis exhibit DNA damage and chromatin condensation during the early stage, followed by plasma membrane blebbing as well as caspase activation without losing cell membrane integrity [9]. Caspase-1 activation leads to the canonical inflammasome-induced pyroptosis pathway. Human caspase-4,5 and murine caspase-11 activation leads to the non-canonical inflammasome-induced pyroptosis pathway [10,11]. The pore-forming domain, the main executor of pyroptosis, found in these caspases is similar to the one found in the crystal structure of the human gasdermin (GSDM) superfamily (GSDMA, GSDMB, GSDMC, GSDMD, DFNA5 and DFNB59). Multiple studied have shown the abnormal expression of the GSDM family in human cancers, which implicates the potential roles in the tumorigenesis and the development. The association between pyroptosis and cancer is complicated. Pyroptosis appears to exert a dual function in cancer progression and treatment. Not only does pyroptosis result in the release of inflammatory factors which stimulate the transformation of normal cells into tumor cells, but it can promote tumor cell death. Pyroptosis plays different roles in many different types of cancer. It may be proved beneficial in preventing colorectal tumor development, and it inhibits tumor growth in hepatocellular carcinoma [12,13]. Recent studies have explored and identified novel pyroptosis-related signatures in some cancers. For example, a pyroptosis-related signature was constructed to predict patient prognosis and response to immunotherapy in gastric cancer [14]. Pyroptosis-related genes also play an important role in tumor immunity and can be used to predict the prognosis of ovarian cancer [15]. A prognostic signature for lung adenocarcinoma was built based on pyroptosis-related regulators [16]. However, the prognostic value of pyroptosis-related genes in THCA has not yet been elucidated.

Therefore, our study was aimed at developing a novel prognostic signature based on pyroptosis-related genes to systematically explore the relationship between the signature and clinicopathological features and overall survival (OS) in THCA patients. Furthermore, tumor immune microenvironment (TIME), mutation profile and the response to ICI treatment associated with the signature in THCA were further explored. The signature could predict the prognosis and immunotherapy response. In addition, this study provides a better understanding of the relationship between pyroptosis and immunotherapy response in THCA patients.

## Materials and methods

### Data collection

A flowchart was illustrated in Additional file 1: Figure S1 to show the research methodology. We downloaded 568 gene expression profiles (58 normal samples and 510 tumor samples) of THCA and OS clinical information from The Cancer Genome Atlas (TCGA) database (https://portal.gdc.cancer.gov/). Patients were randomly divided into a training set (n = 251) and a test set (n = 251) (Additional files 3, 4, 5). There were no significant differences in clinical variables between the two sets (Table 1). A total of thirty-three pyroptosis-related genes were obtained from prior reviews [17–19].

**Table 1:** The clinical characteristics in training, test and total sets

Variables	Type	Total set (n = 502)	Test set (n = 251)	Training set (n = 251)	P value
Age	≤ 60	389 (77.49%)	198 (78.88%)	191 (76.1%)	0.5214
	> 60	113 (22.51%)	53 (21.12%)	60 (23.9%)	
Gender	Female	367 (73.11%)	184 (73.31%)	183 (72.91%)	1
	Male	135 (26.89%)	67 (26.69%)	68 (27.09%)	
Stage	Stage I–II	333 (66.33%)	174 (69.32%)	159 (63.35%)	0.2297
	Stage III–IV	167 (33.27%)	77 (30.68%)	90 (35.86%)	
	Unknow	2 (0.4%)	0 (0%)	2 (0.8%)	
T	T1-2	307 (61.16%)	164 (65.34%)	143 (56.97%)	0.0662
	T3-4	193 (38.45%)	86 (34.26%)	107 (42.63%)	
	Unknow	2 (0.4%)	1 (0.4%)	1 (0.4%)	
M	M0	282 (56.18%)	137 (54.58%)	145 (57.77%)	0.9415
	M1	9 (1.79%)	5 (1.99%)	4 (1.59%)	
	Unknow	211 (42.03%)	109 (43.43%)	102 (40.64%)	
N	N0	229 (45.62%)	112 (44.62%)	117 (46.61%)	0.7067
	N1	223 (44.42%)	114 (45.42%)	109 (43.43%)	
	Unknow	50 (9.96%)	25 (9.96%)	25 (9.96%)	

### Differentially expressed genes (DEGs) identification

We identified DEGs in all tumor and normal samples using the “limma” package in R language (version 4.0.4). A p value of < 0.05 was set as the screening criterion. The DEGs were signed with * if p < 0.05, ** if p < 0.01 and *** if p < 0.001.

### Construction of the protein–protein interaction (PPI) network

A PPI network was constructed using the Search Tool for the Retrieval of Interacting Genes/Proteins (STRING) database (http://www.string-db.org/) to explore the interactions between these DEGs.

### Consensus clustering of pyroptosis-related genes

THCA patients were clustered into different subgroups based on the pyroptosis-related DEGs using the “ConsensusClusterPlus” package in R [20].

### Construction of pyroptosis-related prognostic signature

Patients with THCA were divided into a training set and a test set at a 1:1 ratio. The training set was used to identify prognostic pyroptosis-related genes and develop a prognostic risk signature. The predictive capability was validated in the test set and total set. A univariate Cox proportional hazard regression was employed to identify the pyroptosis-related genes with prognostic values of OS. To prevent omissions, a cut-off p value < 0.2 was set to identify prognostic variables. Subsequently, we used a least absolute shrinkage and selection operator (LASSO) penalized Cox proportional hazards regression to avoid overfitting and constructed the prognostic signature with the “glmnet” package [21]. The model was determined by penalty parameter (λ) with tenfold cross-validation following the minimum criteria (i.e. the value of λ corresponding to the lowest partial likelihood deviance). The risk scores of each THCA patient were calculated based on the gene expression level and its coefficient. The risk score was calculated as follows: risk score = sum (pyroptosis gene expression level × corresponding coefficient). Patients were classified into high- and low-risk groups according to the median risk score. Principal Component Analysis (PCA) and t-distributed Stochastic Neighbor Embedding (t-SNE) were implemented using the “stats” and “Rtsne” packages, respectively. To validate the predictive power, Kaplan–Meier survival curves were analyzed using the “survival” and “survminer” packages and the area under the curves (AUCs) were calculated with the “survivalROC” package [22].

### Functional enrichment analysis

All samples were divided into high- and low-risk groups according to the prognostic signatures. Gene Ontology (GO) and Kyoto Encyclopedia of Genes and Genomes (KEGG) enrichment analyses were conducted using the “clusterProfiler” package in R software according to the DEGs (|log2FC|≥ 1 and FDR < 0.05) between the high- and low-risk groups. Meanwhile, GSEA was performed in the Hallmark gene set “h.all.v7.4.symbols.gmt” to analyze the enriched biological pathways of key genes using GSEA 4.1.0. A NOM p-value of < 0.05 was considered statistically significant.

### Estimation of tumor-infiltrating immune cells

The immunoscore of every patient was obtained from the ESTIMATE algorithm using the “estimate” package [23]. CIBERSORT is a deconvolution algorithm based on RNA-Seq data to estimate the composition ratio of immune cells [24]. We calculated the relative proportions of 21 types of infiltrating immune cells in all tumor samples based on THCA transcriptional profiles. A Wilcoxon rank-sum test was used to evaluate the difference in the level of immune cell infiltration in high- and low-risk groups.

### Evaluation of immune status

Single-sample GSEA (ssGSEA) was used to calculate the scores of 16 infiltrating immune cells and the activity of 13 immune-related pathways in the high- and low-risk groups using the “GSVA” package of R [25]. We also compared the expression of the HLA gene between the high- and low-risk groups.

### Mutation analysis

The mutation data of THCA patients were also obtained from the TCGA data portal (https://portal.gdc.cancer.gov/). The data were further analyzed using the “maftools” package [26]. We calculated the tumor mutation burden (TMB) score of every patient as follows: (total mutation ÷ total covered bases) × 10^6 [27].

### Quantitative PCR

Sixty-five matched tumorous and non-tumorous tissue specimens of PTC were collected from the First Affiliated Hospital of China Medical University. The clinicopathological characteristics of 65 THCA patients from our hospital are displayed in Table 2. Total RNA was extracted from tissue samples using RNAiso (Takara, Dalian, China), then RNA was reverse transcribed into cDNA with the QuantiTect Reverse Transcription Kit (Takara, Shiga, Japan). Quantitative Real-Time PCR (qRT-PCR) analyses were performed with SYBR-Green (Takara, Shiga, Japan) to validate gene expression, and the level of GAPDH served as an internal control. The relative expression was calculated based on the comparative Ct (2^−ΔΔCt^) method [28]. The primers’ sequences are listed in Table 3.

**Table 2:** The clinicopathological features of THCA (N = 65)

Characteristics	Samples (N = 65)	Percentage (%)
Age		
≤ 60	58	89
> 60	7	11
Gender		
Female	46	71
Male	19	29
Tumor size		
< 2 cm	42	65
≥ 2 cm	23	35
Extrathyroidal invasion		
Yes	8	12
No	57	88
Multicentricity		
Yes	26	40
No	39	60
Stage		
Stage I–II	59	91
Stage III–IV	6	9
T		
T1–2	44	68
T3–4	21	32
N		
N0	20	31
N1	45	69

**Table 3:** Premier sequences for qRT-PCR analysis

Premier	Sequences (5′–3′)
PJVK-F	GGAAGGCGAGGTAACCATATTG
PJVK-R	TTCTGCTGCTCCTTGACTGAC
NOD1-F	CGAGACACAGAGCCAGAAGGT
NOD1-R	CGCCGTAGTCGTTGAGATTGTT
IL18-F	AGTTCTCTTCATTGACCAAGGA
IL18-R	CATACCTCTAGGCTGGCTATCT
GAPDH-F	GTCTCCTCTGACTTCAACAGCG
GAPDH-R	ACCACCCTGTTGCTGTAGCCAA

## Results

### Identification of DEGs in the TCGA cohort of THCA

**Fig. 1:**
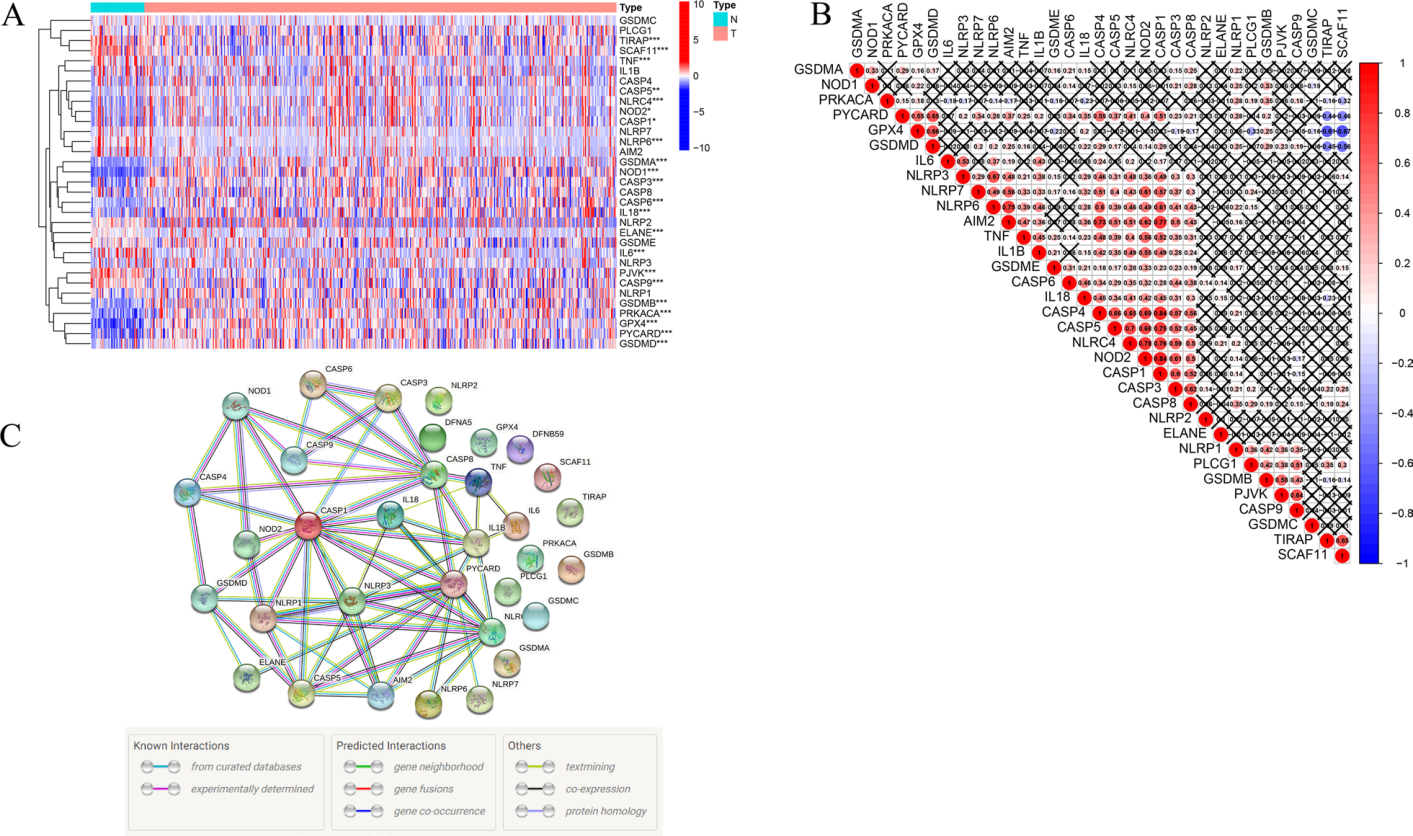
Expression of the pyroptosis-related genes in THCA. **A** The heatmap showed the expression levels of 33 pyroptosis-related genes in normal and tumor samples. *P < 0.05, **P < 0.01, ***P < 0.001. **B** Pearson correlation analysis of the 33 pyroptosis-related genes in THCA. **C** PPI network indicated the interactions of the pyroptosis-related genes

Based on the P-value < 0.05, a total of 22 differentially expressed genes were identified from 33 pyroptosis-related genes. Among them, 15 were up-regulated (*CASP1, CASP3, CASP5, CASP6, GSDMA, GSDMB, GSDMD, NOD1, NOD2, ELANE, NLRC4, PRKACA, GPX4, PYCARD, IL18*) and 7 were down-regulated (*NLRP6, IL6, TNF, PJVK, SCAF11, TIRAP and CASP9*) in THCA tumors. The heatmap shows the RNA expression levels of these genes (Fig. 1A). In addition, we analyzed the correlation among pyroptosis-related genes: GPX4 was significantly negatively correlated with SCAF11 (Cor = − 0.67), while CASP1 and NOD2, and CASP4 were significantly positively correlated (Cor = 0.84) (Fig. 1B). A protein–protein interaction (PPI) network with the minimum required interaction score (highest confidence 0.9) was constructed to further explore the interactions among these pyroptosis-related genes (Fig. 1C).

### Consensus clustering of pyroptosis-related genes

**Fig. 2:**
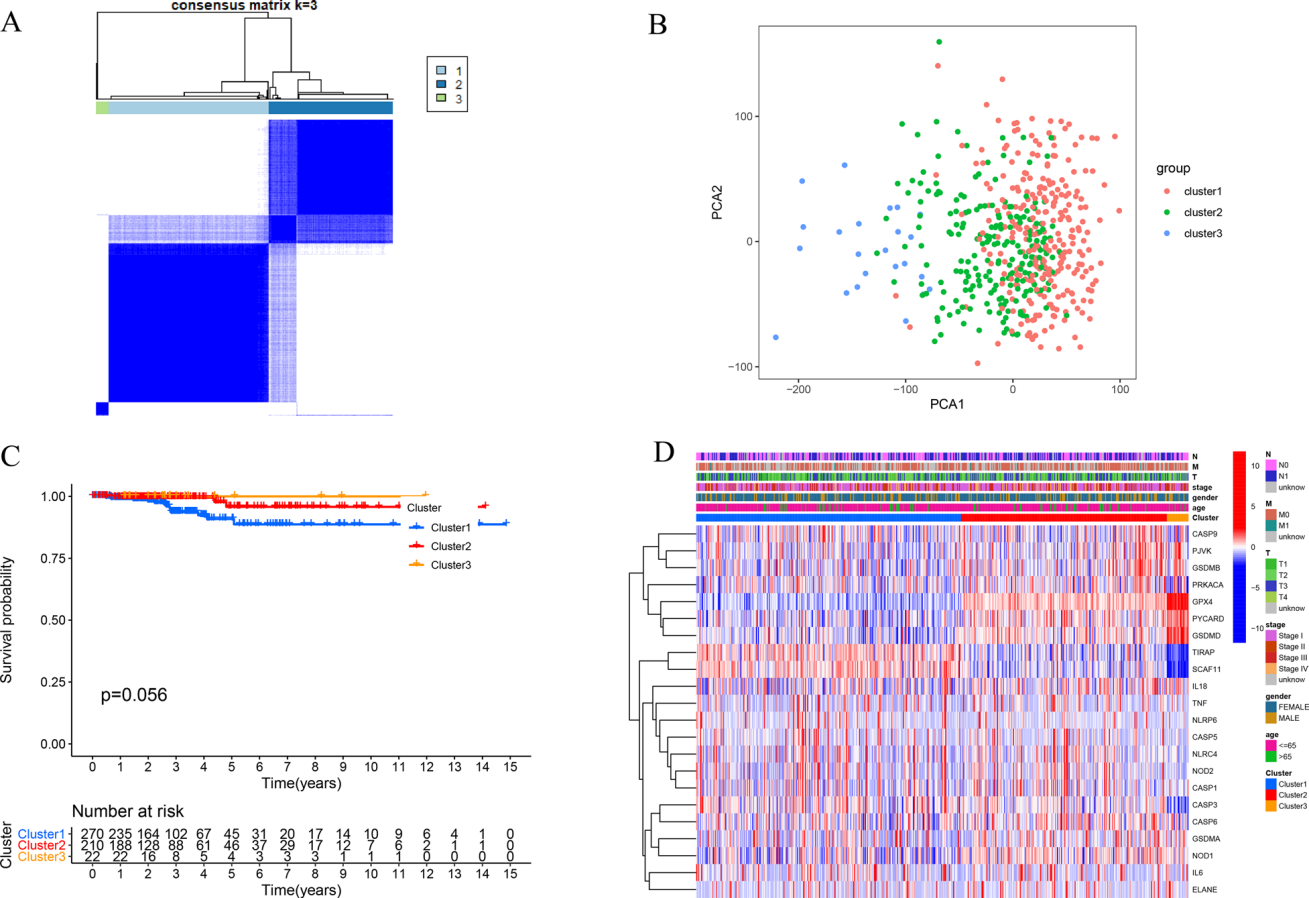
Identification of consensus clusters based on the pyroptosis-related genes. **A** Consensus clustering matrix for k = 3. **B** Principal Component Analysis (PCA) of the RNA expression profile in TCGA cohort. **C** Heatmap and clinicopathologic features of the three clusters. **D** Kaplan–Meier curves of overall survival (OS) in three clusters

In order to investigate the connections between the expression of the pyroptosis-related DEGs and THCA subtypes, we performed a consensus clustering analysis using the ConsensusCluserPlus package based on the 22 pyroptosis-related DEGs. The number of clusters was represented by the letter “k”. The k = 3 was identified with optimal clustering stability from k = 2 to 9. Finally, the THCA patients were clustered into three subtypes, namely, Cluster1 (n = 270), Cluster2 (n = 210) and Cluster3 (n = 22) (Fig. 2A). We also analyzed the gene-expression pattern between three subtypes of PCA. The results suggested that Cluster1, Cluster2 and Cluster3 could gather together (Fig. 2B). There were no statistical differences between the three clusters and OS (Fig. 2C). When the gene expression profile and clinicopathological features between the three subtypes were compared with a heatmap, no significant correlations were found between clinicopathological features in the three subtypes (Fig. 2D).

### Construction of pyroptosis-related risk signature

**Fig. 3:**
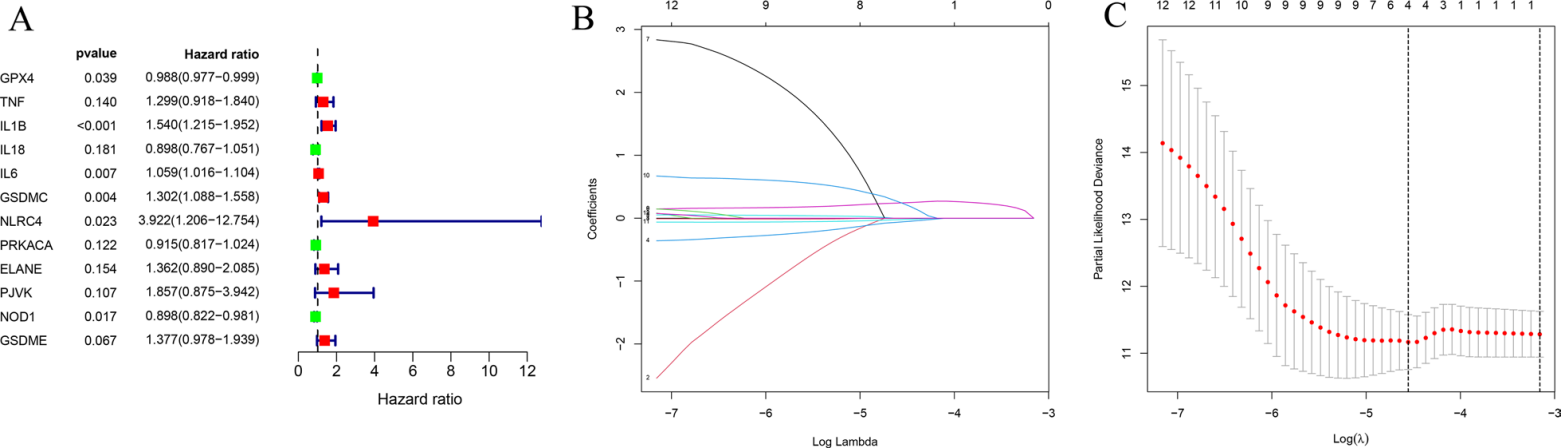
Univariate Cox regression analysis and LASSO analysis. **A** Forest plot showing the result of univariate Cox regression analysis of OS, 12 genes with p < 0.2. **B** Cross-validation for tuning parameter selection in the LASSO regression. **C** LASSO analysis of 12 prognostic pyroptosis-related genes

**Fig. 4:**
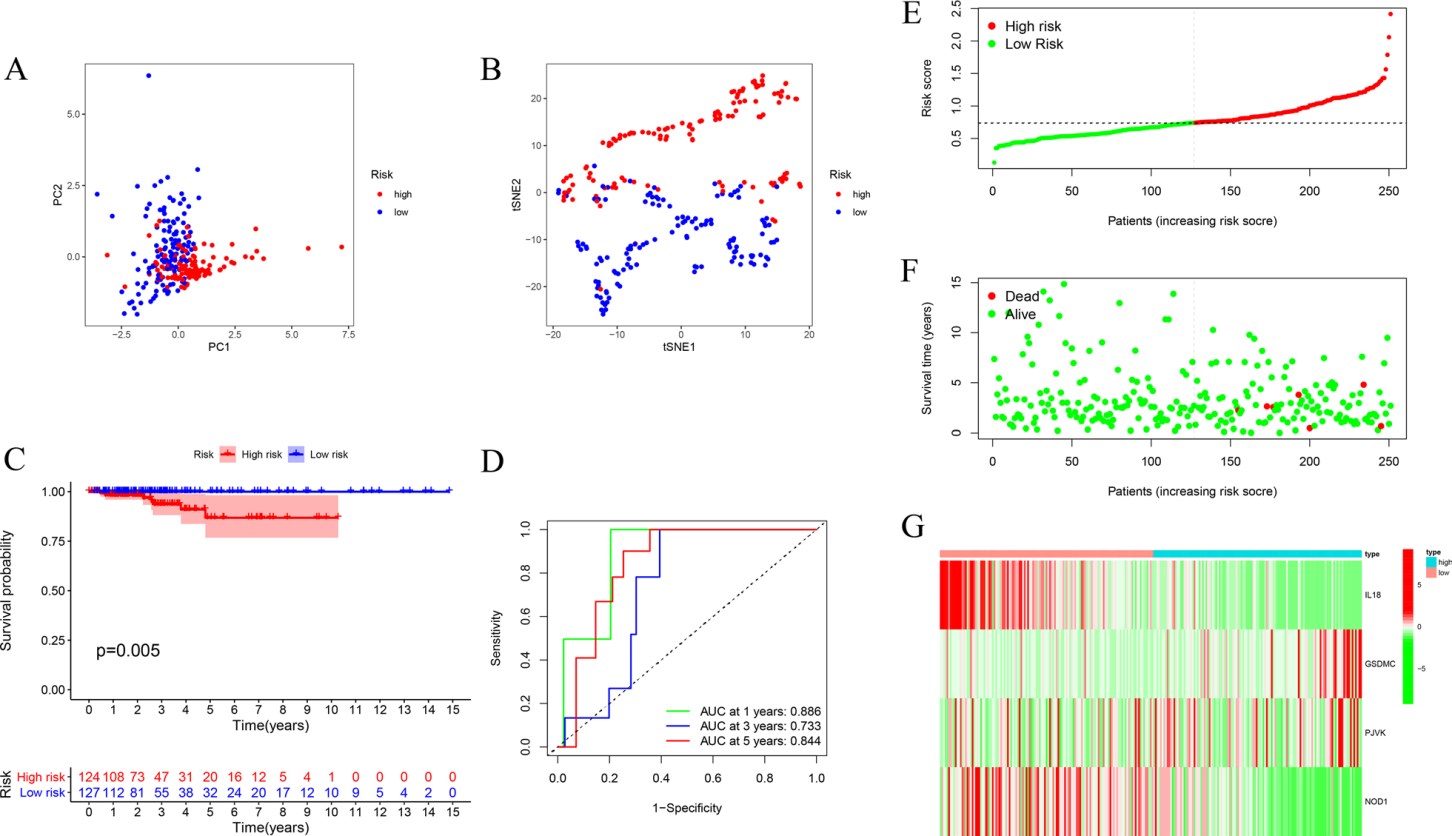
Construction of risk signature in the training set. **A** PCA plot, **B** t-SNE analysis of TCGA cohort in training set. **C** Kaplan–Meier curves for OS of THCA patients in high- and low-risk groups. **D** Time-dependent ROC analysis. **E** The distribution of risk score, (**F**) survival status, and (**G**) the expression patterns of 4 pyroptosis-related genes in high- and low-risk groups

We performed a univariate Cox regression analysis to identify the prognosis-related genes. The 12 genes that met a criterion with P-value < 0.2 were significantly associated with survival in the training set. Among them, 4 genes (GPX4, IL18, PRKACA and NOD1) were protective genes with HR < 1, while 8 genes (TNF, IL1B, IL6, GSDMC, NLRC4, ELANE, PJVK and GSDME) were detrimental genes with HR > 1 (Fig. 3A). To minimize overfitting, the set underwent LASSO Cox regression analysis, and 4 of the 12 genes were chosen to construct a risk signature based on the optimum λ value (Fig. 3B, C). The formula of the four-gene signature was as follows: Risk score = (− 0.059 × IL18) + (0.2427 × GSDMC) + (0.2389 × PJVK) + (− 0.023 × NOD1). Subsequently, all patients with THCA in the training set were classified into the high- and low-risk groups based on the median risk score. PCA and t-SNE indicated that patients with different risks were distributed into two directions (Fig. 4A, B). Patients with high-risk scores had a significantly poorer OS than patients in the low-risk group (p = 0.005) (Fig. 4C). The areas under the curve (AUC) of the risk signature were 0.886 at 1-year, 0.733 at 3-year and 0.844 at 5-year (Fig. 4D). In addition, we ranked the patients’ risk scores and analyzed their distributions in the training set (Fig. 4E). The survival status of THCA patients in the training set was presented in the dot plot (Fig. 4F). The heatmap showed the expression patterns of 4 prognostic genes between two risk groups (Fig. 4G).

**Fig. 5:**
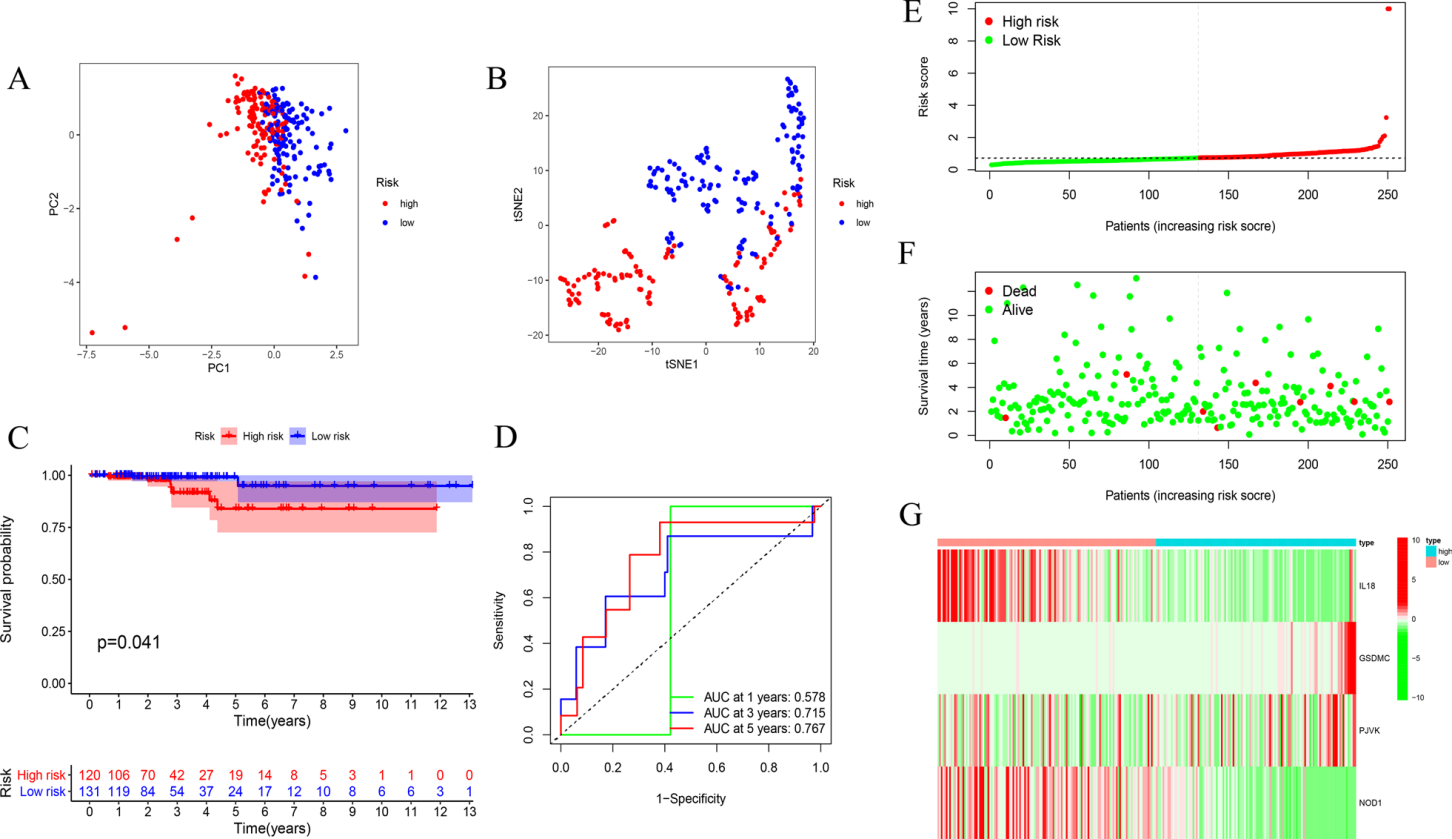
Validating the 4 pyroptosis-related genes risk signature in the testing set. **A** PCA plot, **B** t-SNE analysis for THCA in testing set. **C** Kaplan–Meier curves for OS of THCA patients in high- and low-risk groups. **D** Time-dependent ROC analysis. **E** The distribution of risk score, **F** survival status, and (**G**) the expression patterns of 4 pyroptosis-related genes in high- and low-risk groups

**Fig. 6:**
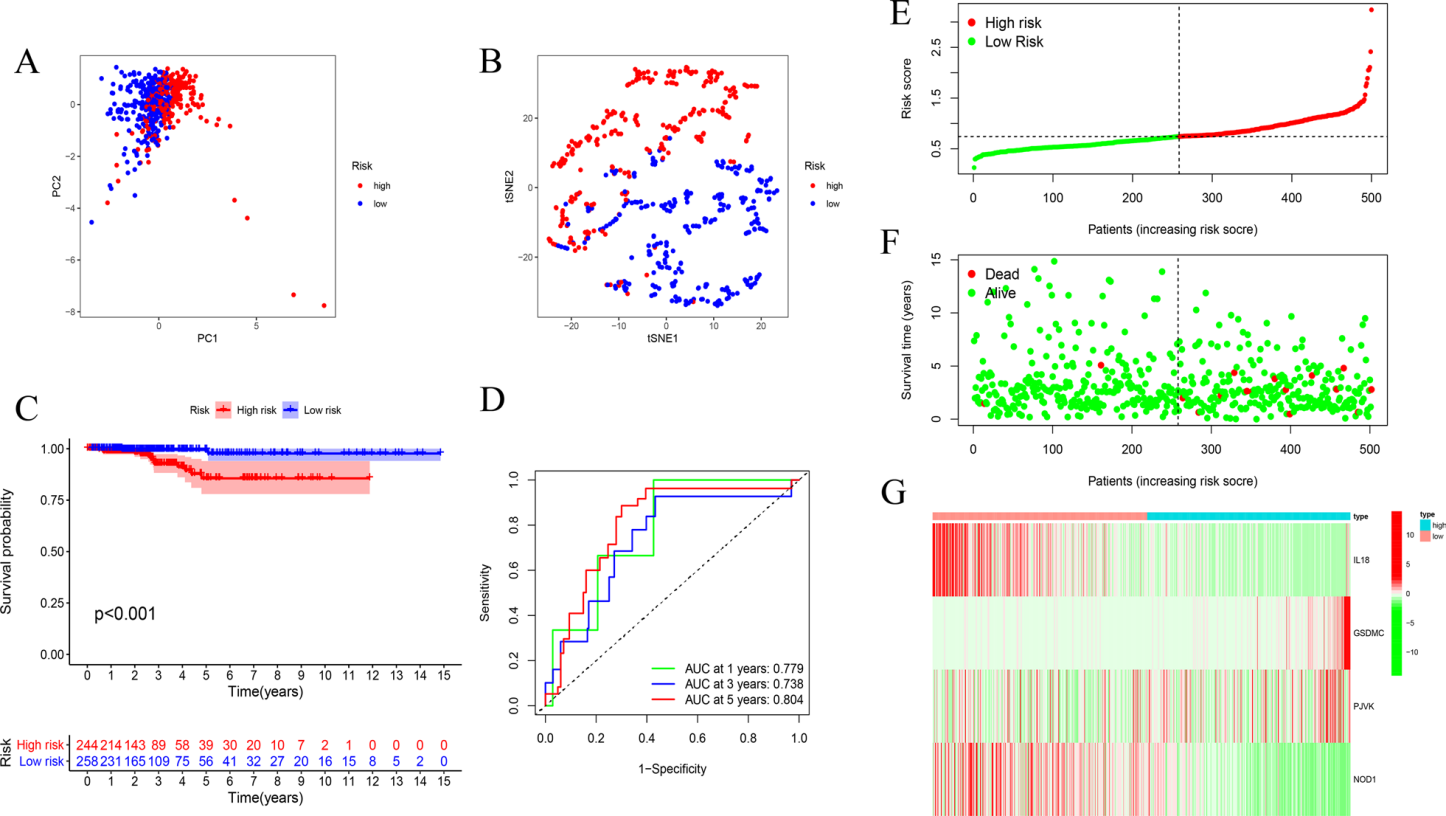
Validating the 4 pyroptosis-related genes risk signature in the total set. **A** PCA plot, (**B**) t-SNE analysis for THCA in total set. **C** Kaplan–Meier curves for OS of THCA patients in high- and low-risk groups. **D** Time-dependent ROC analysis. (E) The distribution of risk score, (**F**) survival status, and (**G**) the expression patterns of 4 pyroptosis-related genes in high- and low-risk groups

Furthermore, the predictive capability of risk signature was verified in the test set and total set. The risk scores of every patient were calculated and the patients were divided into high- and low-risk groups in two sets as previously described. PCA and t-SNE confirmed that the patients in the different subgroups were separated into two clusters (Figs. 5A, B, 6A, B). Kaplan–Meier survival curves indicated that the OS of high-risk patients was lower than that of the low-risk groups in the test group (p = 0.041) (Fig. 5C). The 1-year AUC was 0.578, the 3-year AUC was 0.715 and 5-year AUC was 0.767 (Fig. 5D). The distribution of the risk score, survival status and the expression of 4 pyroptosis-related genes in the test set are presented in Fig. 5E–G.

The results in the total set were similar to those in the training set and test set. The OS was significantly different between the two risk groups (p < 0.001) (Fig. 6C). The AUCs for 1-year, 3-year and 5-year were 0.779, 0.738 and 0.804 (Fig. 6D). The distribution of risk score, patients’ survival status and expression heatmap of 4 prognostic genes are also displayed in Fig. 6E–G.

### Independent prognostic value of the risk signature

**Fig. 7:**
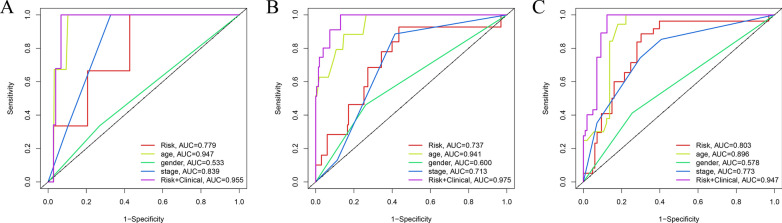
The time-dependent ROC to evaluate the prognostic power based on risk score and clinical factors in (**A**) 1-year, (**B**) 3-year, (**C**) 5-year

A univariate Cox regression analysis was performed to explore the relationship between clinicopathological variables and risk score on OS of THCA patients in the total set (Table 4). The risk score could serve as an independent prognostic factor for THCA in the total set referring to the results of the multivariable Cox regression analysis. Meanwhile, the ROC curve indicated that the AUC increased when combining the risk score with other clinicopathological features, which suggested that the risk score was an independent prognostic factor (Fig. 7A–C).

**Table 4:** Univariate and multivariate Cox regression analyses

Variables	Univariate analysis				Multivariate analysis			
	HR	HR.95L	HR.95H	P	HR	HR.95L	HR.95H	P
Training set								
Age	1.16	1.07	1.25	0.00	1.16	1.06	1.26	0.00
Gender	0.50	0.06	4.15	0.52	0.50	0.06	4.57	0.54
Stage	3.20	1.42	7.18	0.00	2.65	0.91	7.74	0.07
Riskscore	5.58	1.24	25.11	0.03	15.80	1.25	199.28	0.03
Test set								
Age	1.16	1.08	1.26	0.00	1.20	1.08	1.34	0.00
Gender	4.27	1.14	15.98	0.03	3.04	0.49	18.92	0.23
Stage	2.07	1.17	3.66	0.01	0.91	0.39	2.10	0.82
Riskscore	1.15	1.06	1.25	0.00	1.19	1.05	1.36	0.01
Entire set								
Age	1.16	1.10	1.22	0.00	1.17	1.11	1.25	0.00
Gender	1.92	0.69	5.30	0.21	1.09	0.33	3.60	0.88
Stage	2.42	1.53	3.80	0.00	1.49	0.81	2.75	0.20
Riskscore	1.15	1.06	1.24	0.00	1.20	1.09	1.32	0.00

### Risk signature and prognostic analysis of clinicopathological factors

**Fig. 8:**
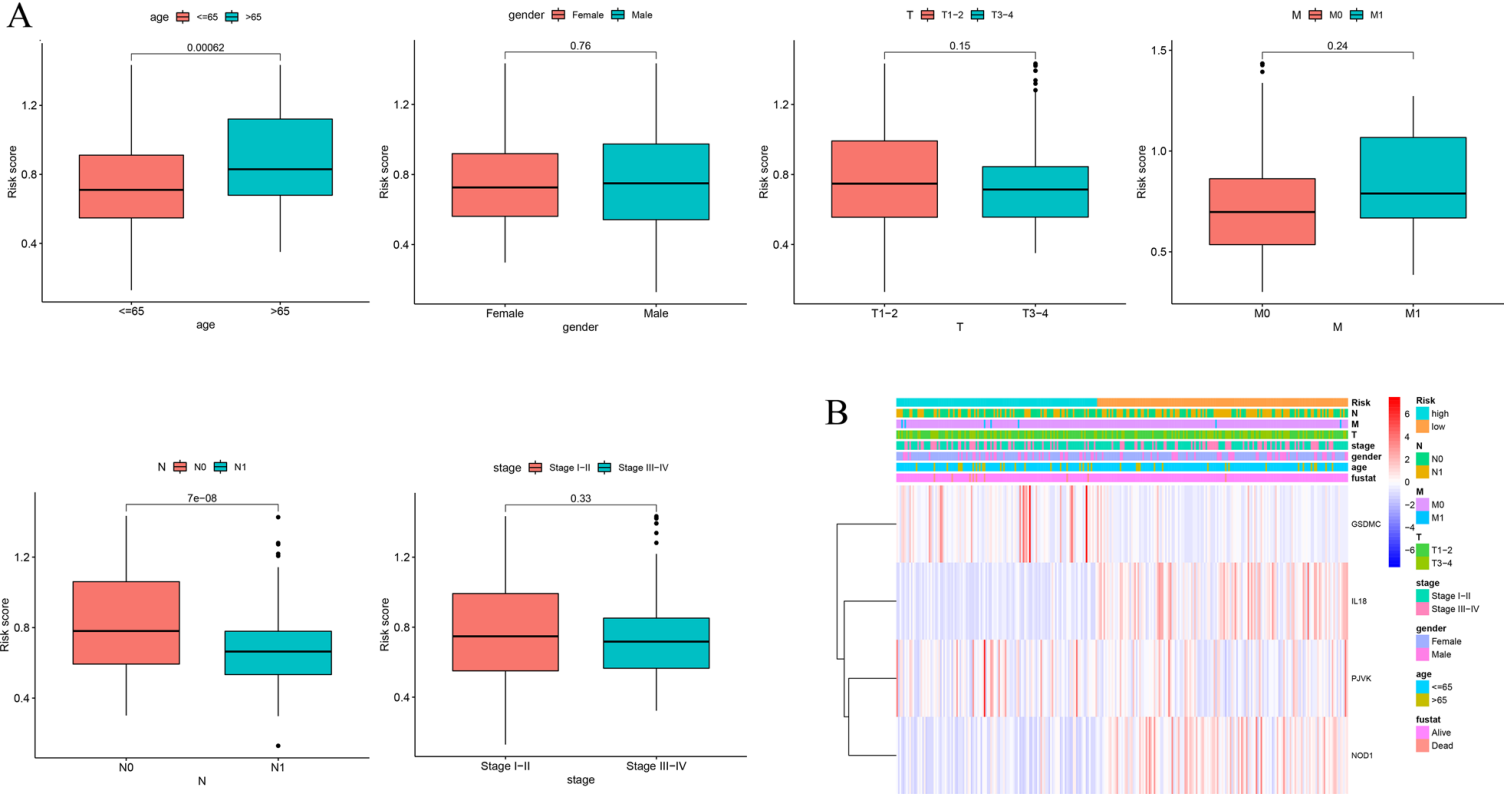
The relationships between risk signature and clinic-pathologic parameters (**A**) (age, gender, T stage, M stage, N stage and clinical stage). **B** Heatmap showing the connections between clinicopathologic factors and high- and low-risk groups

We analyzed the relationships between the risk signature and clinicopathological factors. The results suggested that there were significant differences between the different ages and N stages. The risk score was significantly lower in patients with N1 stage and age below 65. Nevertheless, the risk signature was not correlated with gender, T stage, M stage and clinical stage (Fig. 8A). Additionally, survival analysis showed patients with high-risk scores were inclined to have a poorer OS in all subgroups (Additional file 2: Figure S2). The heatmap displayed the relationship between prognostic gene expression and clinical factors (Fig. 8B).

### Functional analyses based on the risk signature

**Fig. 9:**
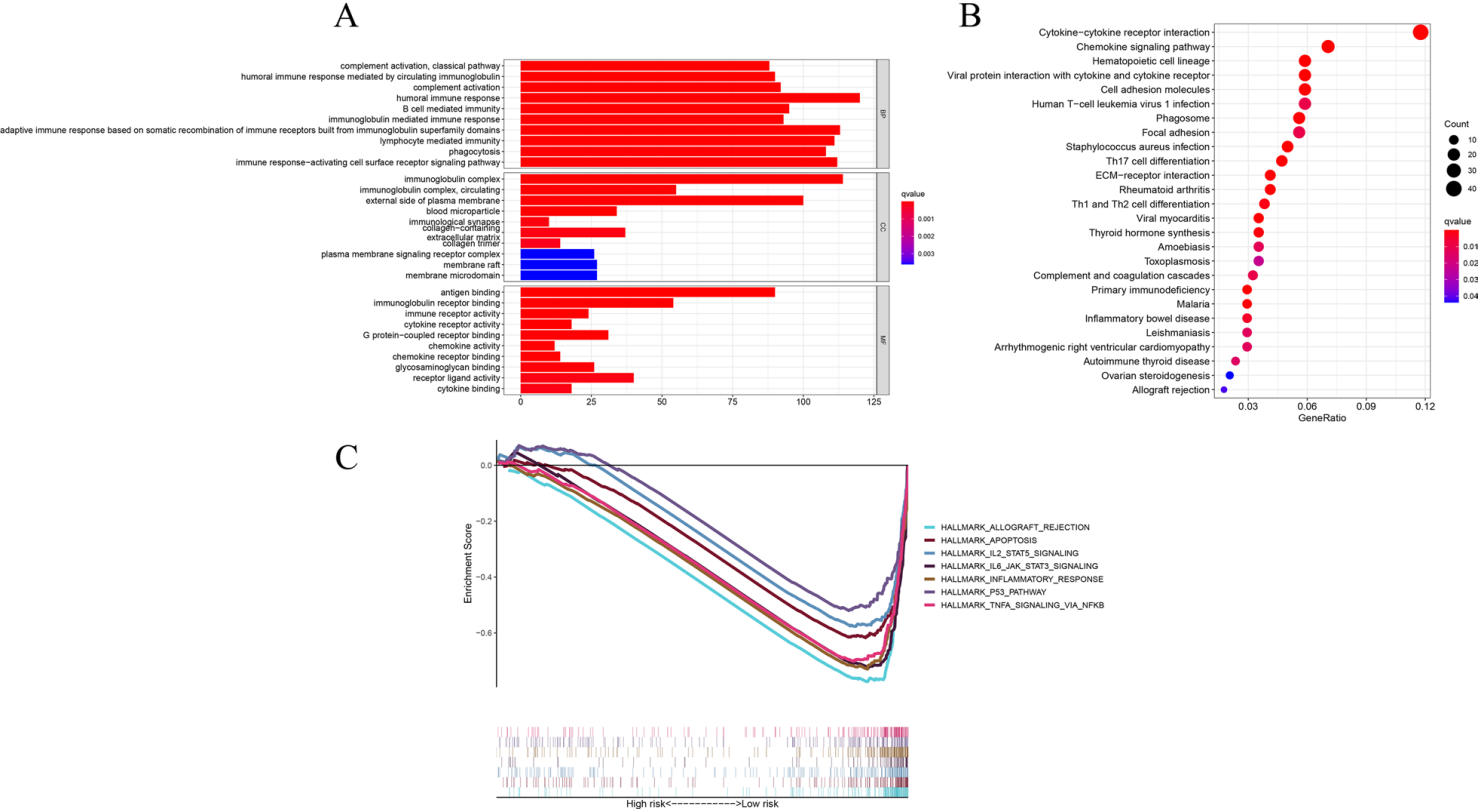
Functional enrichment analysis based on the DEGs. **A** Bar plot graph for GO enrichment. **B** Bubble graph for KEGG pathways. **C** Gene set enrichment analysis (GSEA) showed the significantly enriched hallmarks of tumor sets based on the risk signature in TCGA

To further elucidate the potential biological functions and pathways that are correlated with the risk score, GO enrichment and KEGG pathway analyses were performed according to the DEGs between the high- and low-risk groups. The results suggested that the DEGs were mainly enriched in immune response, cytokine–cytokine receptor interaction and chemokine signaling pathway (Fig. 9A, B).

### Gene set enrichment analyses (GSEA)

The transcript messages of THCA patients classified by risk score into high- and low-risk subgroups were analyzed by GSEA. The results revealed that the majority of pyroptosis-related prognostic signature genes regulate the immune and malignant hallmarks of THCA tumors. Specifically, biological pathways such as allograft rejection, apoptosis, IL2-Stat5 signaling, IL6-Jak-Stat3 signaling, inflammatory response, P53 pathway signaling, and TNFA signaling via NF-κB were found to be enriched in the low-risk group (Fig. 9C).

### Difference of the tumor-infiltrating immune cell populations between high- and low-risk groups

**Fig. 10:**
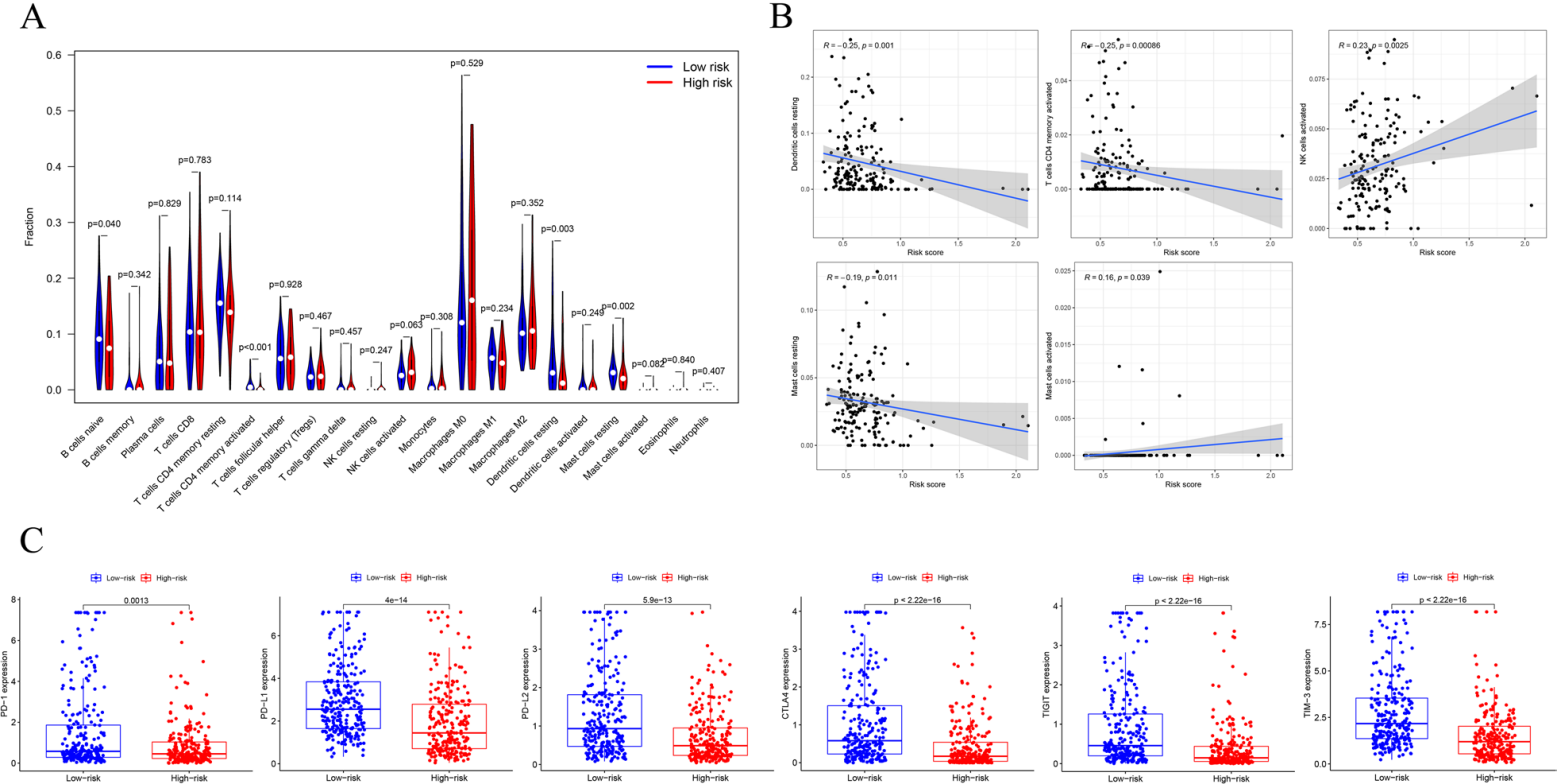
The associations of tumor-infiltrating immune cells and risk scores and immunotherapy gene expression analysis. **A** The infiltrating levels of immune cells in high- and low-risk groups. **B** Correlation between the risk score and infiltration abundances of immune cells. **C** The gene expression of PD-1, PD-L1, PD-L2, CTLA4, TIGIT and TIM-3 in high- and low-risk groups

Immune cells played an important part in the tumor immune microenvironment (TIME) and we analyzed the difference of the tumor-infiltrating immune cell population in the high- and low-risk groups to evaluate the relationship between the prognostic signature and TIME. The CIBERSORT algorithm was utilized to calculate the relative proportion of 22 types of immune cells in each of the THCA patients. The ratios of naive B cells, activated CD4 memory T cells, resting dendritic cells, and resting mast cells were significantly higher in the low-risk group (Fig. 10A). Moreover, the correlation analyses between the risk score and degree of immunocyte infiltration indicated that activated NK cells (R = 0.23, P = 0.0025) and activated mast cells (R = 0.16, P = 0.039) were positively correlated with risk score. However, the risk score was negatively correlated with activated CD4 memory T-cells (R = − 0.25, P = 0.00086), resting dendritic cells (R = − 0.25, P = 0.001), and resting mast cells (R = − 0.19, P = 0.011) (Fig. 10B).

Numerous studies have shown that immunotherapy is emerging as a new hope in cancer treatment, and immune checkpoint proteins play important parts in the immune response. Therefore, we compared the expression levels of common immune checkpoint proteins in the high- and low-risk groups. The results indicated that low-risk patients had significantly higher expression levels of PD-1 (programmed cell death 1), PD-L1 (programmed cell death ligand 1), PD-L2 (programmed cell death ligand 2), CTLA-4 (cytotoxic T-lymphocyte-associated protein 4), TIGIT (T Cell Immunoreceptor with Ig and ITIM Domains) and TIM-3 (T-cell immunoglobulin and mucin-domain containing-3) (p < 0.01) (Fig. 10C). The results indicated that patients with low pyroptosis-related signature score might have a better opportunity for ICI treatment.

### Comparing the immune status between the high- and low-risk groups

**Fig. 11:**
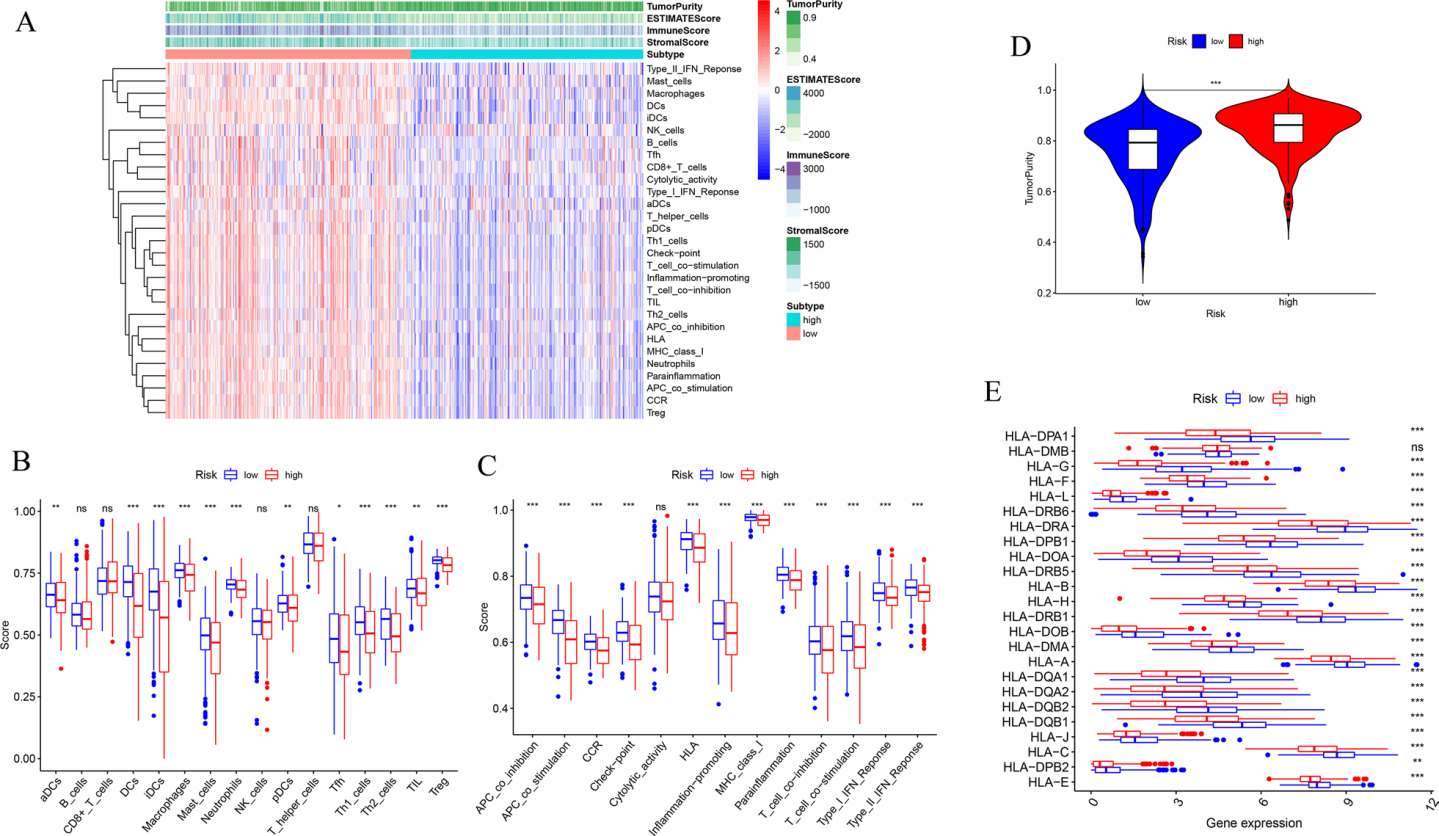
Comparison of the ssGSEA scores in high- and low-risk groups. **A** The immune status, (**D**) tumor purity and (**E**) the expression of HLA related genes in high- and low-risk groups. The boxplot showed the enrichment scores of (**B**) 16 immune cells and (**C**) 13 immune-related functions between high- and low-risk groups

To further evaluate the relationship between the immune status and the risk score, we quantified the infiltrating scores of 16 immune cells and the activity of 13 immune-related pathways between the high- and low-risk groups in the TCGA cohort of THCA using single-sample gene set enrichment analysis (ssGSEA). The heatmap shows the immune status of 29 immune signature gene sets in the high- and low-risk groups (Fig. 11A). The scores of aDCs, DCs, iDCs, macrophages, mast cells, neutrophils, pDCs, Tfh, Th1 cells, Th2 cells, TIL and Treg were significantly different between the two subgroups (Fig. 11B). Except for the cytolytic activity pathway, the immune-related pathways had higher activity in the low-risk group than in the high-risk group (Fig. 11C). The low-risk group showed not only more immune activities, but significantly lower tumor purity (Fig. 11D). Additionally, we analyzed the expression levels of HLA related genes. The results suggested that the low-risk group had higher expression levels of HLA genes compared to those in the high-risk group (Fig. 11E).

### A pyroptosis-related risk signature and mutation profile

**Fig. 12:**
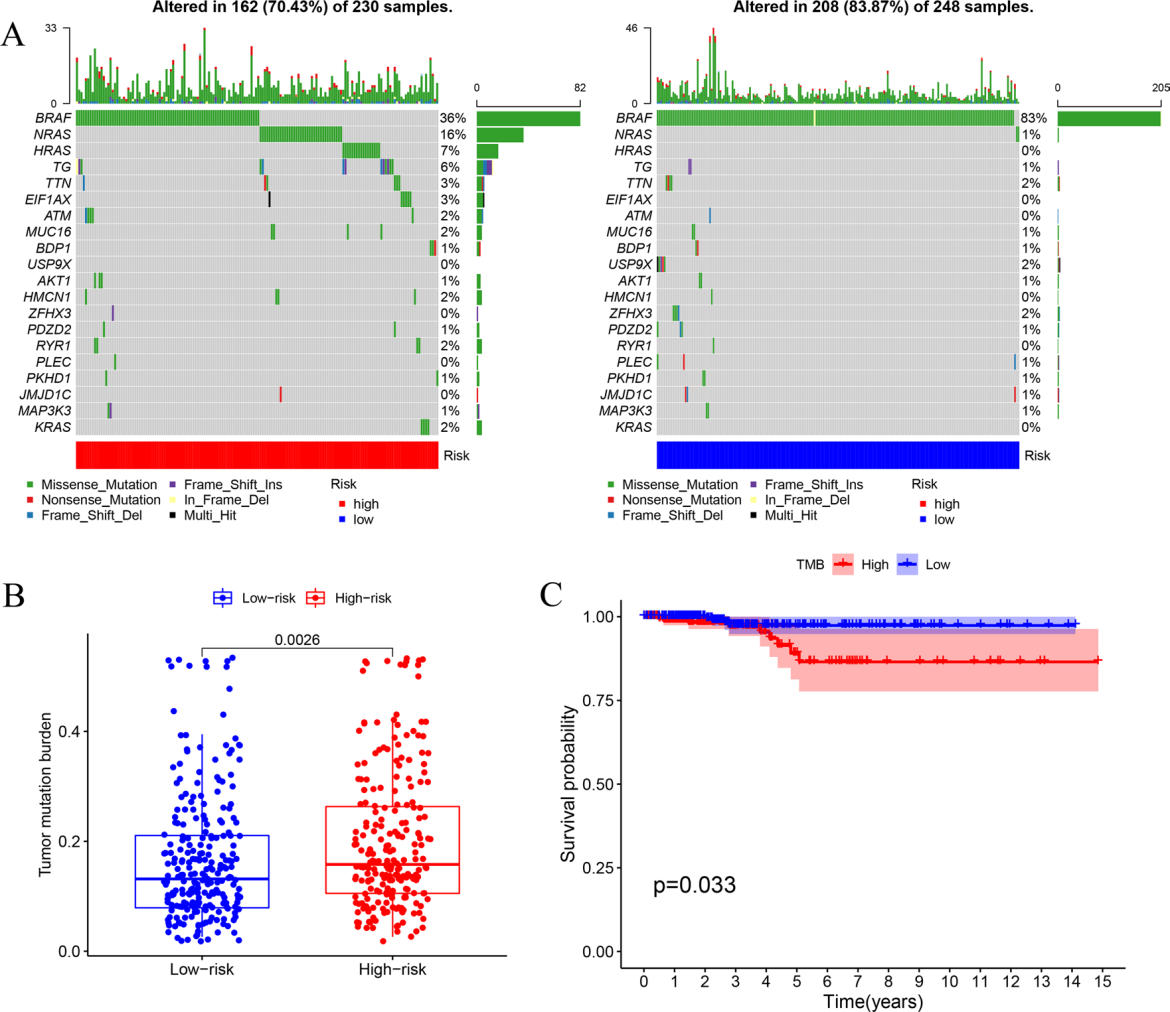
The mutation profile and TMB in high- and low-risk groups. **A** Mutation profile of THCA patients in high- and low-risk groups. **B** The relationship between the risk signature and TMB. **C** The association between TMB and OS

Gene mutation is one of the significant factors in tumorigenesis and development. We evaluated the relationship between the signature and mutation profile in THCA patients. The top three mutated genes in THCA patients were BRAF, NRAS and HRAS. The most frequently mutated genes in the high- and low-risk groups are shown in Fig. 12A. The level of TMB was markedly higher in the high-risk group than that in the low-risk group (p = 0.0026) (Fig. 12B). Furthermore, we observed that TMB was associated with OS (p = 0.033) (Fig. 12C).

### The expression levels of four prognostic genes

**Fig. 13:**
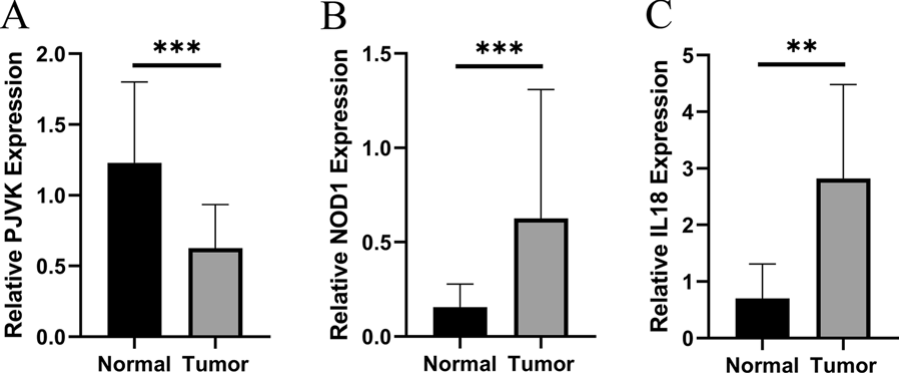
The expression levels of PJVK (**A**), NOD1 (**B**) and IL18 (**C**) quantified using qRT-PCR analysis in 65 paired thyroid cancer tissues and no-tumorous samples

We further validated the expression of the four prognostic genes (IL18, GSDMC, PJVK and NOD1) in 65 pairs of clinical samples from patients with PTC using qRT-PCR analysis according to the bioinformatics analysis results. The results of the qRT-PCR showed that the mRNA expression of NOD1 and IL18 were significantly higher in PTC tissues (p < 0.05). However, the expression of PJVK was decreased in PTC samples (p < 0.001) (Fig. 13), which was consistent with the results of bioinformatic analysis.

## Discussion

THCA is the most common endocrine malignancy and PTC accounts for more than 85% of all thyroid cancer cases. Thyroid nodules are very common. Ultrasound guided fine needle aspiration (US-FNA) is the most commonly available way to evaluate thyroid nodules. However, rapid On-Site Evaluation (ROSE) is found to be the most helpful with small sized nodules or nodules that are more difficult to sample for less experienced radiologists [2]. Patients with PTC have a > 90% 10-year survival rate after proper surgical treatment and radiotherapy [29]. In radiotherapy, we must consider harmful effects of radiation for normal tissues surrounding tumor tissues. Accurate calculation of out-of-field dose to be critical for informing risk estimates, such as estimation of out-of-field dose variation using Markus ionization chamber detector [30]. Although the incidence of PTC has increased significantly over the past few decades, the presence of LNM can lead to locoregional recurrence and mortality. Therefore, exploring novel therapeutic targets of THCA is still a major challenging issue.

Recently, the possible beneficial effects of cancer therapies promoting pyroptosis have attracted considerable attention. Pyroptosis, an inflammatory form of programmed cell death, influences the proliferation, invasion and metastasis of tumor cells. It is a more recently identified pathway of programmed cell death that is stimulated by a range of microbial infections and non-infectious stimuli [31]. Pyroptosis is regulated via a caspase-1-dependent or caspase-1-independent mechanism [32]. Pyroptosis exerts a dual function in cancer progression and treatment mechanisms [16]. It plays a vital role in cellular lysis and release of pro-inflammatory cytokines when a host defends against infections [33]. Pyroptosis results in the release of intracellular proinflammatory contents and induces an inflammatory response leading to the death of adjacent healthy cells, which contributes to the development and progression of malignancies [9]. Additionally, pyroptosis can promote tumor cell death which makes pyrolysis a potential novel therapeutic target for cancer treatment. However, the potential role of pyroptosis-related genes in THCA remains unknown. Therefore, we aimed to discover potential diagnostic markers for targeted therapy of pyroptosis to improve the survival of patients with THCA as well as explore the prognostic and diagnostic value of pyroptosis. Our study suggested that using immunotherapy to induce pyroptosis may be an effective therapeutic direction to improve patient prognosis.

In our study, we analyzed the mRNA expression patterns of 33 pyroptosis-related genes in THCA samples and normal samples and 22 were differentially expressed. Among these genes, 15 were up-regulated and 7 were down-regulated. We identified three subgroups of THCA using consensus clustering analysis according to the expression of pyroptosis-related genes and no significant differences were found in the clinicopathological features. We then derived four prognostic risk signatures from these pyroptosis-related genes based on the univariate Cox regression analysis and LASSO Cox regression analysis. The prognostic value of the four prognostic-relevant risk signatures was evaluated in THCA patients and validated using a TCGA internal dataset. Interleukin (IL)-18, belonging to the IL-1 superfamily, is a proinflammatory and immune regulatory cytokine. IL-18 was originally identified as an interferon (IFN)-γ-inducing factor and involved in Th1 and Th2 responses in T cells, natural killer (NK) cells and macrophages [34]. IL-18 plays a dual role in cancer, as it promotes tumor development, progression and metastasis and it enhances anti-tumor immunity and reduces tumor growth in a matter depending on cancer progression [35]. IL-18 in combination with IL-12, through the activation of NK and cytotoxic T-cells, produced IFN-γ, which contributed to tumor immunity and had anti-tumor activity in different preclinical models. At present, IL-18 has been studied as a novel treatment approach and immune checkpoint therapy to significantly improved cancer treatment. Thus, IL-18 combined with immune-checkpoint therapy might be a potential treatment for early-stage tumors [36, 37]. The human gasdermin (GSDM) family (GSDMA, GSDMB, GSDMC, GSDMD, DFNA5 and DFNB59), which modulates multifunctional signal processes, can regulate cell pyroptosis [38]. The abnormal expression of the GSDM family in human cancers has been previously demonstrated, which implies their potential roles in tumorigenesis [39]. Multiple studies have shown that dysregulation of GSDMC expression is correlated with the biological processes of multiple cancers. Saeki et al. [38] found that GSDMC inhibition of tumor cell growth behaved like a potential tumor suppressor in the gastrointestinal epithelium. Watabe et al. [40] proved that GSDMC overexpression promoted tumor cells metastasis and proliferation in B16 melanoma cells. We found that high GSDMC expression was associated with poorer survival, which suggested that GSDMC might participate in tumor cell tumorigenesis and progression in thyroid cancer. PJVK, also called DFNB59, lacks the C-terminal domains. All members of the GSDM superfamily except for PJVK have a complete two-domain structure [41]. We regarded PJVK as a pyroptosis-related gene with a complete N-terminal domain and a similar pore-forming activity to other GSDMs [15]. PJVK has been found expressed in heart, brain and kidney, however, the regulatory roles of PJVK are not well understand [42]. At present, PJVK was demonstrated to be associated with hearing impairment in humans and is located on chromosome 2 [43]. We found high PJVK expression in tumor tissues from patients with a relatively poor prognosis. The nucleotide-binding oligomerization domain protein-1 (NOD1), one of the most important members of the NOD-like receptor (NLR) family, can induce pro-inflammatory responses and is involved in the apoptotic signaling pathway in some tumor cells [44]. Several studies have indicated that NOD1 plays an important role in the development and progression of gastric cancer, colon cancer, breast cancer and cervical cancer [45, 46]. NOD1 was downregulated in tumor samples in our study and its high expression predicted better survival, which suggested that the NOD1 may be a tumor suppressor gene. The risk score was calculated from the risk signatures and classified the patients into high- and low-risk groups. Kaplan–Meier survival curves showed that the OS of high-risk patients was lower than that of the low-risk groups. The AUC of the ROC curve showed the risk signature was efficient in predicting survival prognosis. Univariate and multivariate Cox regression analyses indicated that the risk score was not only an independent risk factor for prognosis, but could predict the clinical characteristics of THCA. The functional enrichment analyses indicated that immune-related pathways were significantly enriched in the low-risk groups. Therefore, we reasonably speculated that the cell pyroptosis could participate in the TIME. To elucidate the association between infiltrating immune cells and THCA, we estimated the infiltration of tumor immune cells between high- and low-risk groups of patients and found that the high-risk groups had higher proportions of naive B-cells naïve, activated CD4 memory T-cells, resting dendritic cells, and resting mast cells, while the low-risk groups’ scores were positively correlated with the proportions of activated NK cells and activated mast cells. Patients with low-risk scores had higher overall immune activity based on the ssGSEA analysis. The tumor purity was significantly enriched in the high-risk group, which suggested the lower infiltration of stromal and immune cells. Recently, cancer immunotherapy has gained wide acceptance as a potential therapeutic agent or an alternative to standard chemotherapy and has made great progress in the field of cancer therapy [47, 48]. Therefore, we explored the response of common immune checkpoints inhibitors and the expression of PD-1, PD-L1, PD-L2, CTLA4, TIGIT and TIM-3 increased significantly in low-risk patients. Our study suggested that patients with low-risk score had higher expression of common immune checkpoint molecules. According to above findings, we speculate that low-risk patients might have a better, more beneficial response from treatment with checkpoint inhibitors of PD-1, PD-L1, PD-L2, CTLA4, TIGIT and TIM-3. TMB was a significant independent predictor of the responses to immunotherapy in diverse cancers. In this study, the predictive value of the risk signature is independent of TMB. We found that the low-risk groups showed lower TMB.

However, at present, there are very few studies of pyroptosis in the thyroid cancer field, and investigation of the potential mechanisms may be meaningful in the future. The exploration of prognostic value of pyroptosis-related genes sets the stage for future mechanism research. Our study still has some limitations that need to be considered. All analyses were performed using a small TCGA cohort, a larger external validation cohort should be used to verify the predictive power of the signature in THCA, preferably validated using GEO datasets. Unfortunately, no survival information of THCA could be obtained from the GEO cohort. Furthermore, some basic experiments are also required to investigate the correlation between the model and the tumor microenvironment.

## Conclusion

Taken together, we constructed a pyroptosis-related prognostic signature of genes that possessed predictive power based on a comprehensive analysis of RNA sequencing data and clinical data of THCA available in TCGA database. The signature was significantly associated with the tumor immunity. This study provided a better understanding of the relationship between pyroptosis and immunotherapy response in THCA patients. The pyroptosis-related signature could provide new possibilities to predict the prognosis and contribute to the development of new individualized therapeutic strategy for future studies of patients with THCA.

## Supplementary Information

**Additional file 1**: **Figure S1**: The flowchart of the study.

**Additional file 2**: **Figure S2**: The stratification analysis of clinical factors based on age, gender, TNM stage and clinical stage.

**Additional file 3**. The data of the total set.

**Additional file 4**. The data of training set.

**Additional file 5**. The data of test set.

## Data Availability

Gene expression profiles, clinical information and mutation data of THCA in this study are available from the public database (TCGA, https://portal.gdc.cancer.gov/).
