# Supplementary figures and images for: Identification and validation of a pyroptosis-related prognostic signature for thyroid cancer
Source: Cancer Cell Int. 2021 Oct 9;21:523. doi: 10.1186/s12935-021-02231-0 (PMC8502398; doi:10.1186/s12935-021-02231-0)

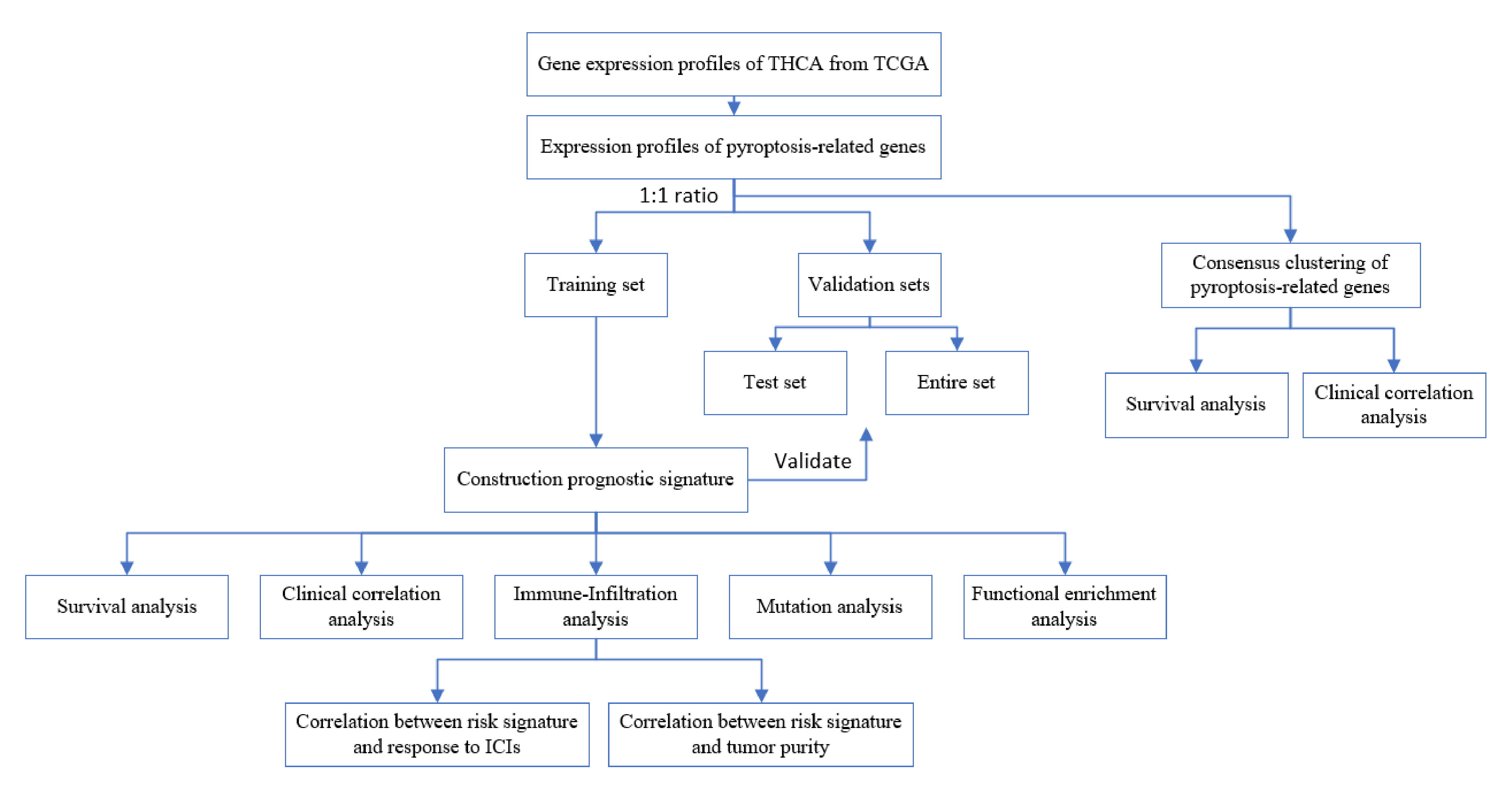

Supplement: Supplementary file 1 — Additional file 1: Figure S1: The flowchart of the study. [file 12935_2021_2231_MOESM1_ESM.tif]

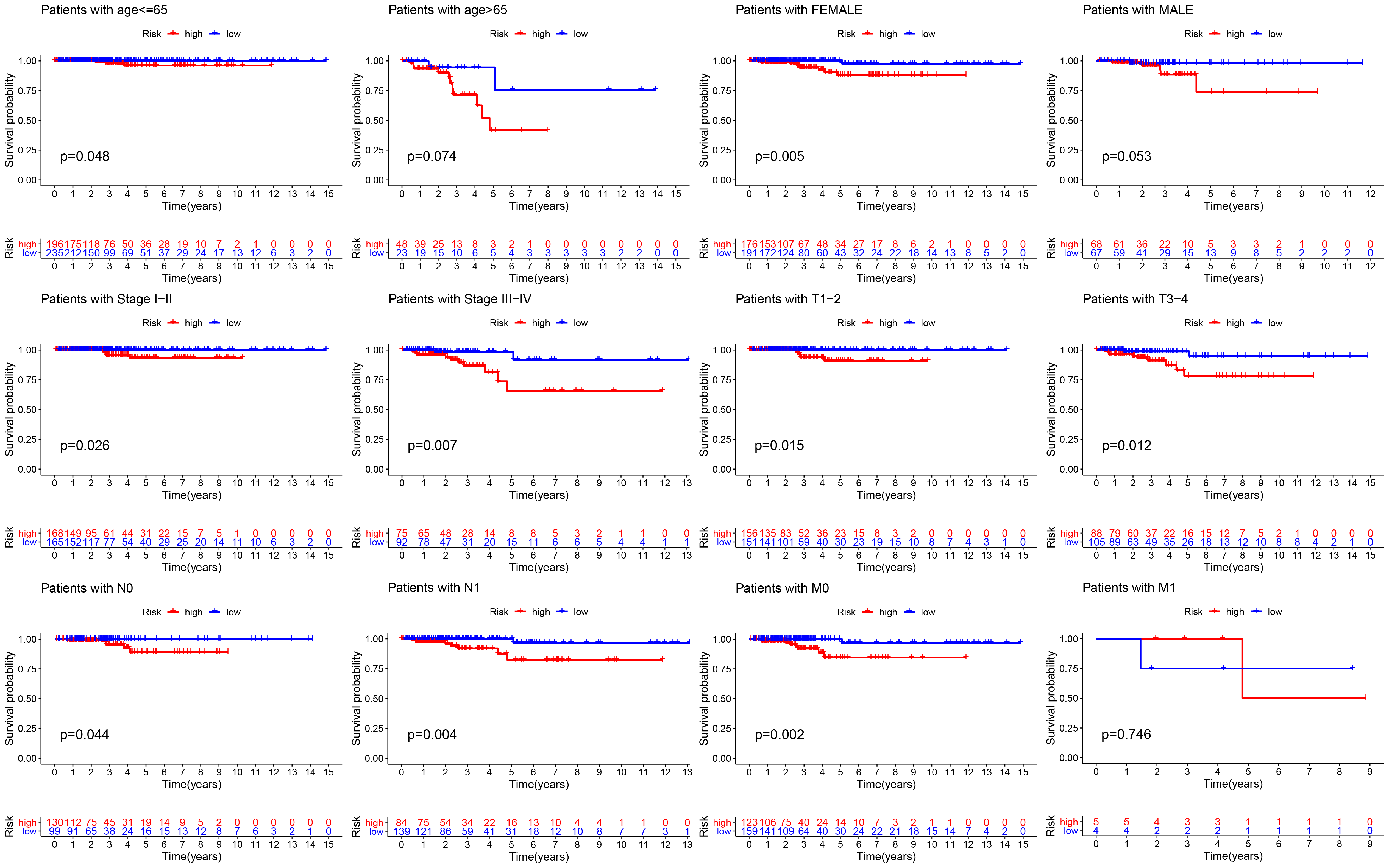

Supplement: Supplementary file 2 — Additional file 2: Figure S2: The stratification analysis of clinical factors based on age, gender, TNM stage and clinical stage. [file 12935_2021_2231_MOESM2_ESM.tif]
